# Neovascular PSMA expression is a common feature in malignant neoplasms of the thyroid

**DOI:** 10.18632/oncotarget.23984

**Published:** 2018-01-04

**Authors:** Birthe Heitkötter, Konrad Steinestel, Marcel Trautmann, Inga Grünewald, Peter Barth, Heidrun Gevensleben, Martin Bögemann, Eva Wardelmann, Wolfgang Hartmann, Kambiz Rahbar, Sebastian Huss

**Affiliations:** ^1^ Gerhard Domagk Institute of Pathology, University of Münster, Münster, Germany; ^2^ Institute of Pathology, Military Hospital Ulm, Ulm, Germany; ^3^ Institute of Pathology, Neuss, Germany; ^4^ Department of Urology, University Hospital Münster, University of Münster, Münster, Germany; ^5^ Department of Nuclear Medicine, University Hospital Münster, Münster, Germany

**Keywords:** PSMA, thyroid cancer, tumor neoangiogenesis, prostate cancer

## Abstract

**Aim:**

PSMA (prostate-specific membrane antigen) is physiologically expressed in normal prostate tissue and over expressed in prostate cancer cells, therefore constituting a potential target for antibody-based radioligand therapy. Very recent imaging findings reported PSMA-PET/CT uptake in various thyroid lesions. We were therefore encouraged to systematically analyse PSMA expression in different benign and malignant thyroid lesions.

**Methods:**

Immunohistochemistry was used to detect PSMA expression in 101 thyroid lesions, while neovasculature was identified by CD34 immunostaining.

**Results:**

PSMA expression in the neovasculature was significantly more frequent in malignant tumors (36/63; 57.1%) compared to benign diseases (5/38; 13.2%; *p* = 0.0001). In addition, PSMA expression levels in the neovasculature of poorly and undifferentiated thyroid cancers were significantly higher compared to differentiated thyroid tumors (*p* = 0.021). However, one case with a strong expression in follicular adenoma was identified.

**Conclusions:**

We conclude that neovascular PSMA expression is common in thyroid cancer but may also rarely be found in benign thyroid diseases, such as follicular adenoma. High expression in the tumor-associated neovasculature is predominantly found in poorly differentiated and undifferentiated (anaplastic) thyroid cancer. This knowledge is highly relevant when interpreting PSMA/PET-CT scans from patients with prostate cancer. In addition, our findings might provide a rationale for further evaluation of PSMA-targeted anti-neovascular or radioligand therapy in metastatic dedifferentiated thyroid cancer.

## INTRODUCTION

PSMA (prostate specific membrane antigen) is a 100 kDa type II-transmembrane glycoprotein with both intra- and extracellular protein domains exerting folate hydrolase and neurocarboxypeptidase activity [1, 2]. It was originally reported to be physiologically expressed by prostate cells [3, 4].A Strong upregulation in prostate cancer cells, which was further described, led to the development of PSMA-based imaging technologies for the detection of metastatic disease in advanced prostate cancer. PSMA-based radioligand therapy has furthermore been established as a therapeutic regimen in metastatic prostate cancer [2, 5–11]. In addition, PSMA was found to be expressed in the endothelium of tumor-associated neovasculature in some solid malignancies (breast, lung and urothelial cancer), possibly due to the effect of tumor-associated angiogenic factors [12–16].

Functional studies revealed that the role of PSMA in tumor angiogenesis is part of an autoregulatory loop involving β1-integrin and p21-activated kinase 1 (PAK1). In this context, active PSMA facilitates endothelial cell invasion through the extracellular matrix by interacting with the cytoskeleton via integrin signaling and actin-binding protein Filamin A [7, 17].

Very recently, PSMA expression has also been reported in thyroid cancer [18–20]. The authors of these studies described the detection of differentiated follicular and papillary thyroid cancer using PSMA-PET/CT Imaging. As thyroid cancer has not been systematically analysed for PSMA expression, the current study aims at investigating different entities of thyroid malignancies, including papillary, follicular, medullary and undifferentiated (anaplastic) thyroid carcinomas. We also included benign and inflammatory lesions, such as adenomas and thyroiditis as well as sporadic nodular goiter, since Gordon *et al.* recently reported increased PSMA expression in nonneoplastic, regenerative and reparative neovasculature [7].

## RESULTS

### PSMA expression in benign thyroid diseases

In a patient with biopsy-confirmed acinar adenocarcinoma of the prostate (Gleason score: 4+5 = 9; grade group 5 (ISUP)) ^68^Ga-PSMA PET/CT revealed bone metastases, nodal involvement and a nodule in the left thyroid lobe showing a focal uptake. The thyroid lesion was regarded as being suspicious for thyroid cancer, as metastases to the thyroid are extremely uncommon in prostate cancer patients. To rule out a second malignancy, hemithyroidectomy was performed. However, the nodule was classified as benign thyroid adenoma. A strong (neo-) vascular PSMA expression was noted, providing a good explanation for the observed ^68^Ga-PSMA PET/CT uptake.

**Figure 1:**
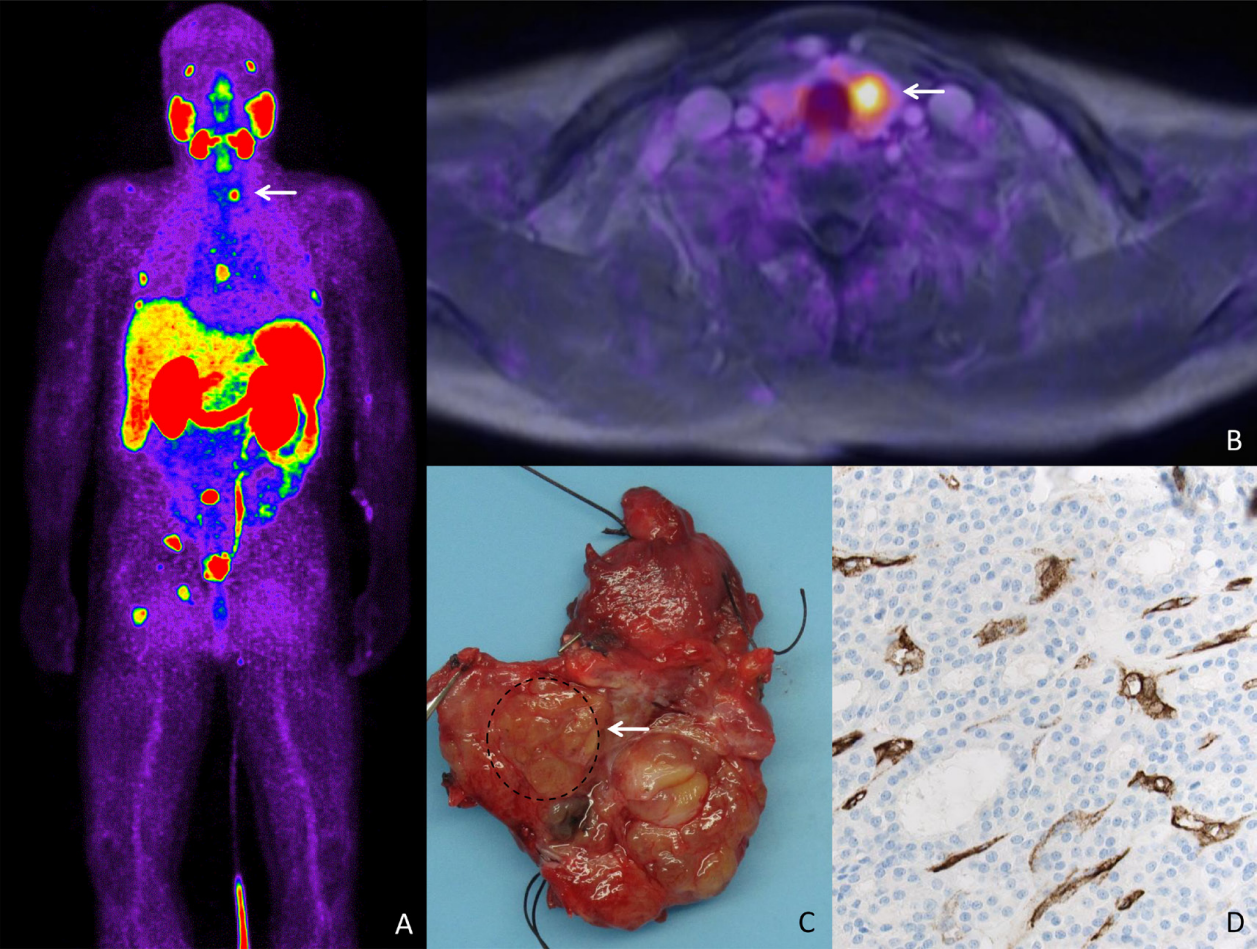
PSMA expression in a patient with a metastatic prostate cancer and thyroid adenoma (**A**) Bodyscan of PSMA-Pet/CT revealing uptake in several bone lesions in a patient with metastatic prostate cancer. Focal uptake is seen in the left thyroid lobe (arrow). (**B**) Transaxial image of fused 68 Ga-PSMA-PET/MRI showing focal uptake in a nodule in the left thyroid lobe. (**C**) Gross section of the thyroid lobe revealing a nodule that was histologically classified as adenoma. (**D**) Strong PSMA expression (200x, IHC) is shown in the adenoma-associated vasculature, apparently being responsible for the PSMA-PET/MRI uptake.

**Figure 2:**
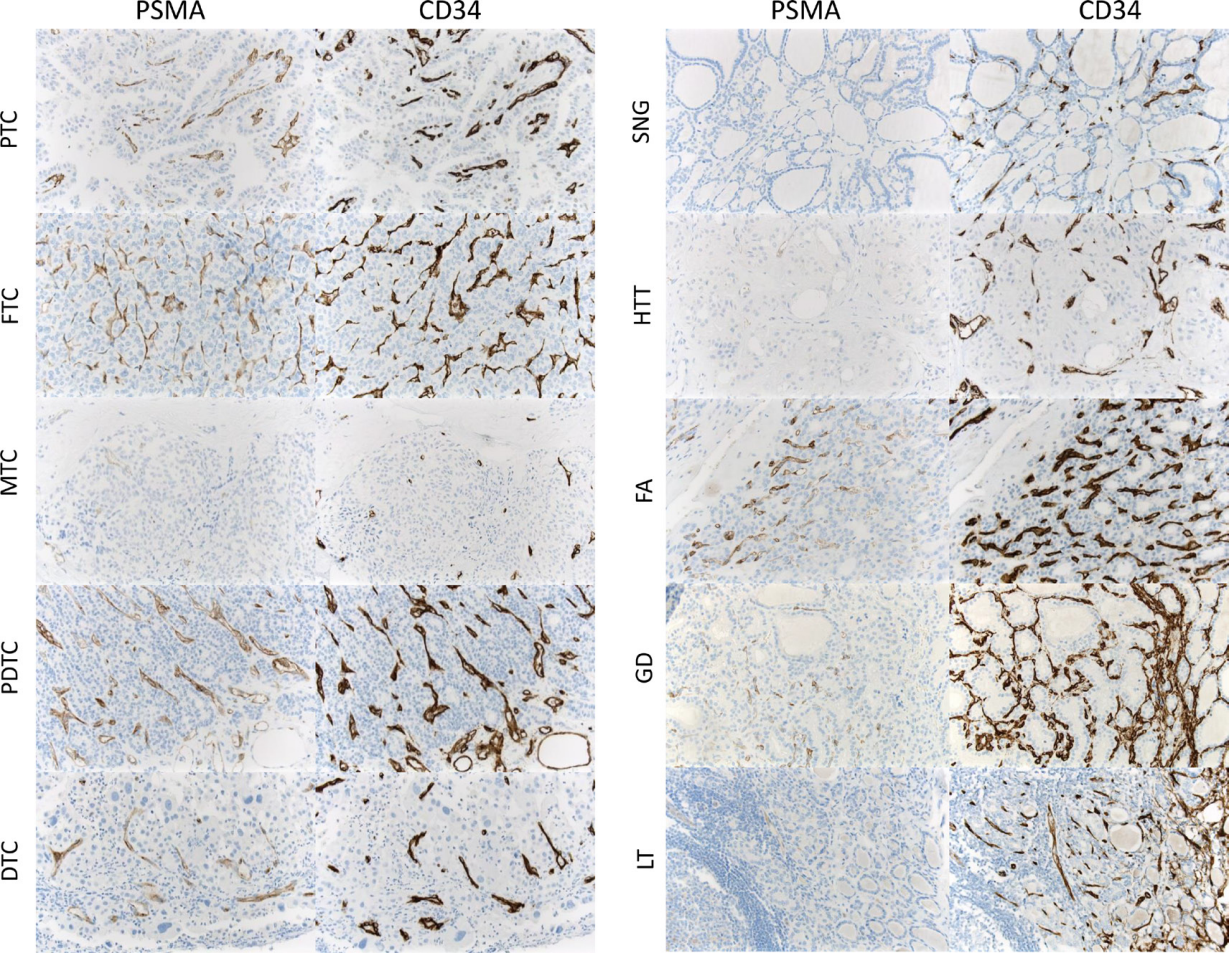
PSMA expression in malignant tumors and benign thyroid diseases (Neo-)vascular PSMA expression in different thyroid cancer subtypes and benign thyroid disease (PTC, papillary thyroid cancer; FTC: follicular thyroid cancer; MTC, medullary thyroid cancer; PDTC, poorly differentiated thyroid cancer; DTC, dedifferentiated (anaplastic) thyroid cancer; SNG, sporadic nodular goiter; FA, follicular adenoma; HTT, hyalinising trabecular thyroid tumor; GD, Grave´s disease; LT, lymphocytic thyroiditis). Vasculature was identified by means of CD31 and CD34 coexpression.

In our immunohistochemical assessment, PSMA expression in the neovasculature was found to be positive in 5/38 benign thyroid diseases (13.2%): 0/18 Sporadic nodular goiter, 2/9 follicular adenoma, 1/2 hyalinising trabecular thyroid tumor, 1/3 Grave´s Disease, 1/2 Lymphocytic thyroiditis, 0/1 Granulomatous thyroiditis, 0/3 unspecific thyroiditis. The majority of these cases (4/38, 10.5%) presented with low expression levels, while the patient diagnosed with follicular adenoma showed a strong PSMA expression (Table 1 and Figures 1 and 2).

**Table 1:** Cases with strong PSMA expression in the (neo-) vasculature (PSMA labelling index = 2; total *n* =19)

Biological Potential	*n*	Cases
Malignant	19	9/31 Papillary thyroid carcinoma 2/10 Follicular thyroid carcinoma 0/12 Medullary thyroid carcinoma 4/6 Poorly differentiated thyroid carcinoma 4/4 Dedifferentiated (anaplastic) thyroid carcinoma
Benign	1	0/18 Sporadic nodular goiter 1/9 Follicular Adenoma 0/2 Hyalinising trabecular thyroid tumor 0/3 Grave´s Disease 0/2 Lymphocytic thyroiditis 0/1 Granulomatous thyroiditis 0/3 Unspecific thyroiditis

### PSMA expression in thyroid cancer

PSMA expression of the tumor-associated neovasculature was found in 36/63 malignant thyroid tumors (57.1%): 18/31 papillary thyroid carcinoma, 4/10 follicular carcinoma, 4/12 medullary carcinoma, 6/6 poorly-differentiated thyroid carcinoma and 4/4 anaplastic thyroid carcinoma. In these malignant tumors, low expression levels could be observed in 9/31 papillary, 2/10 follicular, 4/12 medullary and 2/6 poorly-differentiated thyroid carcinomas (17/63; 37.0%), whereas strong PSMA expression of the neovasculature was noted in nine papillary, two follicular, four poorly-differentiated and four anaplastic carcinomas (19/63; 30.2%) (Table 1 and Figure 2).

**Figure 3:**
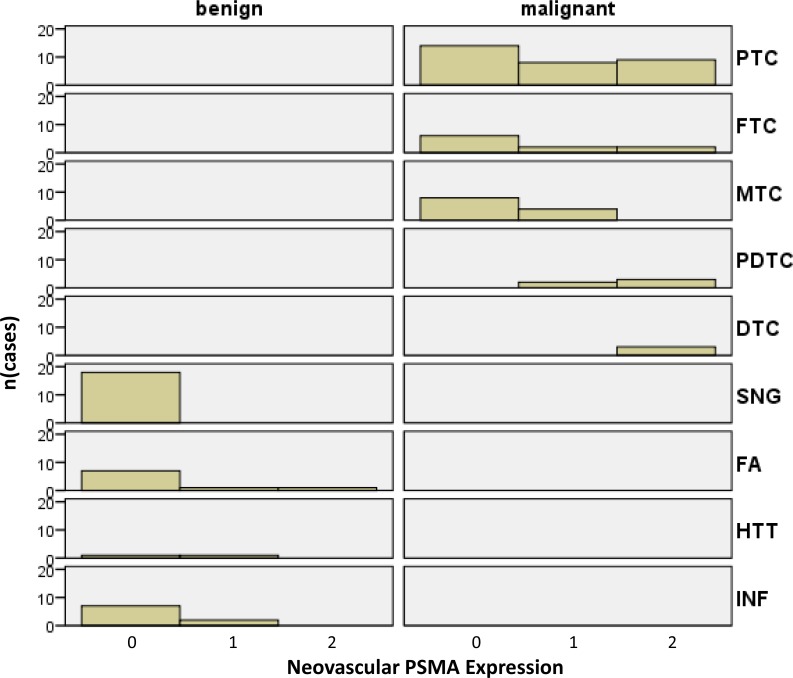
Histograms of malignant tumors and benign thyroid diseases according to their biological potential and PSMA labelling index Histograms of malignant tumors and benign thyroid diseases according to their biological potential and PSMA labelling index (PTC, papillary thyroid cancer; FTC: follicular thyroid cancer; MTC, medullary thyroid cancer; PDTC, poorly differentiated thyroid cancer; DTC, dedifferentiated (anaplastic) thyroid cancer; SNG, sporadic nodular goiter; FA, follicular adenoma; HTT, hyalinising trabecular thyroid tumor; INF, inflammatory diseases).

We then performed a histogram analysis linking the biological potential of the different thyroid diseases to the neovascular PSMA labelling index (Figure 3). The overall PSMA expression (low and high expression, labelling index 1 and 2) was found in the neovasculature of 36/63 (57.1%) malignant tumors, whereas overall PSMA expression in the vasculature of benign diseases (5/38; 13.2%) was significantly lower (*p* = 0.0001, *Fisher´s exact test*).

Analysing only diseases with strong neovascular PSMA expression (labelling index 2), malignant tumors presenting with a high PSMA expression were more frequent (19/63; 30.2%) compared to benign diseases (1/38; 2.63%) (*p* = 0.0006, *Fisher´s exact test*).

Focusing only on the PSMA expression in the neovasculature of malignant thyroid diseases, we noted that the neovasculature of undifferentiated thyroid cancers (poorly-differentiated and anaplastic carcinoma) showed strong PSMA expression in 8/10 (80.00%) cases, while high neovascular PSMA was only rarely observed in differentiated carcinomas (papillary, follicular and medullary thyroid cancers) (11/53 or 20.8% of cases; *p* = 0.0006, *Fisher´s exact test*). Detailed data on staining intensity and histological distribution patterns for each evaluated specimen is shown in Supplementary Table 1.

## DISCUSSION

Apart from its known strong expression in prostatic epithelium and prostate cancer cells, PSMA is expressed in the tumor neovasculature of different tumor types, including renal cell carcinomas, bladder carcinoma, colonic adenocarcinomas, glioblastoma multiforme, lung cancers, malignant melanomas and soft tissue tumors [12–14, 21–23].

Very recently, PSMA uptake in patients undergoing PSMA PET/CT has been reported in cases of thyroid cancer, such as papillary and follicular carcinoma [18, 19]. In our present study, we report on a patient with metastatic prostate cancer, where ^68^Ga-PSMA PET/CT revealed a nodule in the left thyroid lobe with a focal uptake being suspicious for thyroid cancer. After hemithyroidectomy and histopathological workup, the nodule was classified as benign thyroid adenoma. A strong (neo-) vascular PSMA expression was noted, providing a good explanation for the observed ^68^Ga-PSMA PET/CT uptake. This finding prompted us to systematically analyse different thyroid carcinomas for neovascular as well as intratumoral PSMA expression. We included samples of benign thyroid disease, such as sporadic nodular goiter, follicular adenoma and samples of thyroiditis. We expected to find PSMA expression here as well, as Gordon *et al.* reported PSMA expression in nonneoplastic, regenerative, and reparative neovasculature [7]. Indeed, we found PSMA expression in a subset of benign thyroid diseases. However, PSMA expression was observed significantly more often in the neovasculature of malignant thyroid tumors (36/63; 57.1%) compared to the vasculature of benign diseases (5/38; 13.2%; *p* = 0.0001). As expected, our initial patient with follicular adenoma of the thyroid revealed high PSMA expression that was also visible on PSMA PET/CT scan. A similar case has also recently been reported in the literature by others [24]. Very recently, Bychkov *et al.* investigated PSMA expression in thyroid tumors and reported comparable results. The authors describe neovascular PSMA expression in more than 50% of their thyroid cancer cases. They furthermore recognized microvascular PSMA expression in 19% of follicular adenomas [25].

In our cohort, PSMA expression in the neovasculature of poorly and undifferentiated thyroid cancer was shown to be significantly higher than in differentiated thyroid cancer (*p* = 0.021). The biological significance of this finding is unclear but might be due to intratumoral hypoxia as a result of rapid growth of these neoplasms. Since PSMA facilitates endothelial cell invasion during angiogenic sprouting, PSMA upregulation may enhance tumor vascularization, supporting tumor growth by provision of oxygen and nutrients [7, 17]. Therefore, targeting PSMA-expressing neovessels might represent a promising therapeutic option in rapidly growing solid tumors. In our study we used an anti-PSMA antibody (subclone 3E6) targeting the extracellular region of PSMA. In contrast to other subclones, this might be better used to predict the likelihood of success of PSMA imaging and therapy. For patients with metastatic prostate cancer, PSMA-targeted radionuclide therapy has been established as a therapeutic and diagnostic option [5, 26]. PSMA-617, which was developed by the German Cancer Research Centre (DKFZ) in Heidelberg, seems to be a promising ligand for diagnostics and therapy of prostate cancer metastases and recurrences. Different studies using Lutetium-177 labeled PSMA-617 in patients with metastatic prostate cancer have shown convincing response rates and acceptable toxicity profiles [6, 27]. Future studies therefore need to investigate possible anti-angiogenic effects of PSMA-targeted radionuclide therapy in tumors with PSMA-expressing neovessels especially in metastatic dedifferentiated thyroid cancer, where radioactive iodine therapy becomes ineffective to control disease. Two recent studies have evaluated PSMA-targeted therapies in different solid cancer subtypes. Milowsky *et al.* recently showed that tumor-associated neovasculature in multiple advanced metastatic solid cancers could be targeted with an Indium-111- labeled PSMA antibody [28]. Another phase I study using the PSMA-targeted docetaxel-containing nanoparticle BIND-014 in patients with advanced solid tumors was recently performed [29].

Our finding of neovascular PSMA expression in thyroid carcinomas might point towards a potential therapeutic use of PSMA-targeted radioligands or antibody-based antiangiogenic agents, especially when tumors dedifferentiate and iodine-based radiotherapy becomes ineffective to control disease. In line with these results, strong PSMA expression was noted in poorly and undifferentiated thyroid carcinomas. However, future studies have to evaluate internalization and retention of radiolabeled PSMA ligands in metastatic dedifferentiated thyroid cancer for a possible radioligand therapy in these patients. In addition, our study reveals that neovascular PSMA expression can be observed in both malignant and benign thyroid diseases. This knowledge is important for pathologists, clinicians and radiologists when interpreting PSMA/PET-CT scans from patients with prostate cancer, as this finding is apparently responsible for tracer uptake that must not been misinterpreted as prostate cancer metastasis.

## MATERIALS AND METHODS

### Cases

A total of 101 cases with different thyroid diseases were selected by two experienced pathologists (Table 2), and whole sections (one per case) from paraffin-embedded formalin-fixed tissues were stained with PSMA, CD34 and CD31. One patient with biopsy-confirmed prostate cancer was included. ^68^Ga-PSMA-PET/CT was performed to rule out metastases. The indication for ^68^Ga-PSMA-imaging was appointed by an interdisciplinary tumor board, and the patient gave informed consent to the use of his data in this study. The study was approved by the local ethics committee (Az. 2016-445-f-S).

**Table 2:** Malignant neoplasms and benign diseases included in the present study (*n* = 101)

Biological Potential	Cases
Malignant	Papillary thyroid carcinoma (31) Follicular thyroid carcinoma (10) Medullary thyroid carcinoma (12) Poorly differentiated thyroid carcinoma (6) Dedifferentiated (anaplastic) thyroid carcinoma (4)
Benign	Sporadic nodular goiter (18) Follicular Adenoma (9) Hyalinising trabecular thyroid tumor (2) Grave´s Disease (3) Lymphocytic thyroiditis (2) Granulomatous thyroiditis (1) Unspecific thyroiditis (3)

### Immunohistochemistry

Immunhistochemistry (IHC) was performed on 4-µm-thick paraffin sections using the peroxidase-conjugated avidin-biotin method. Antibodies included a monoclonal mouse anti-PSMA antibody (clone 3E6, Ventana, Germany, 1:50 dilution), monoclonal anti-CD34 antibody (clone QBEnd10, Ventana, Germany, ready to use concentration of 0.8µg/ml) and anti-CD31 antibody (clone JC70, Cell Marque, United States, concentration of 0.61µg/ml). Several subclones of anti-PSMA antibodies have been evaluated and commonly used subclones include 7E11 and 3E6. We decided to use clone 3E6 as it targets an extracellular epitope of PSMA. For imaging or therapeutic purposes only antibodies targeting extracellular domains can be potentially used as they have to be internalized by living cells.

In brief, sections were deparaffinized in xylene and rehydrated through graded ethanol at room temperature. Incubation with the primary antibodies was performed for 30 minutes at room temperature. After washing, the sections were incubated with biotinylated secondary antibodies. Immunoreactions were visualized using a 3-amino-9-ethylcarbazole as a substrate (Ventana Optiview DAB IHC detection KIT, Ref: 760–700, Germany). Prostate carcinoma tissue sections served as a positive control.

### Assessment of PSMA expression

PSMA expression was evaluated by two experienced pathologists (BH and KS) on immunostained whole slides. In malignant diseases, tumor cells and associated neovascular endothelium were analysed separately, and the identity of vascular structures was confirmed by CD31 and CD34 coexpression, a common marker for endothelial cells [30–32]. In benign diseases, PSMA expression was analysed in preexistent vascular capillary structures.

In malignant diseases, any reactivity in either tumor cells or tumor-associated vessels was considered positive. Staining intensity was scored semiquantitatively as negative (0), weak (1 = barely perceptible staining at high power (400x) magnification), moderate (2 = readily apparent at low power (40x) magnification) or strong (3). The fraction of PSMA positive cells was assessed as < 5% or > 5%. In the case of heterogeneous staining, the predominant pattern was recorded. For further analysis, labelling indices were defined. A weak (1) or moderate (2) staining intensity in < 5% of the neovasculature and a weak (1) staining intensity in > 5% of the neovasculature was allocated to the “low expression” group (PSMA labelling index = 1), whereas a moderate (2) staining intensity in > 5% of the neovasculature and a strong (3) staining intensity in < or > 5% of the neovasculature were assigned to the “strong expression” group (PSMA labelling index = 2). In benign diseases, PSMA expression was studied in benign tissue and the preexisting vessels using the algorithms described above. This scoring system has been previously established in soft tissue tumors [23, 33].

### ^68^Ga-PSMA-PET/CT

Whole-body PET/CT was performed 65.1 ± 7.0 min after injection of 161 ± 19.8 MBq (range, 131–193 MBq) of ^68^Ga-PSMA-HBED-CC (HBED-CC is *N,N*′-bis [2-hydroxy-5(carboxyethyl)benzyl]ethylenediamine-*N,N*′-diacetic acid) [26]. The patient was asked to void immediately before undergoing scanning. The scans were obtained using a high-resolution hybrid PET/CT system (Biograph mCT, with a 128-slice CT component; Siemens Medical Solutions). Low-dose CT of the entire area covered by PET (from skull to mid-thigh) was performed for attenuation correction. After completion of the CT scan, PET data were acquired for 3 min per bed position. PET images were reconstructed using the standard manufacturer-supplied software (PET resolution of 3 mm).

### Statistics

SPSS-21 software (IBM, Armonk, NY, USA) was used. Fisher’s exact test was used if appropriate. All tests were two-sided with a 95% confidence interval. A *p*-value of ≤5% was considered statistically significant.

## SUPPLEMENTARY MATERIALS TABLE





## Abbreviations

DTC: dedifferentiated (anaplastic) thyroid cancer
FA: follicular adenoma
FTC: follicular thyroid cancer
GD: Grave´s disease
GT: granulomatous thyroiditis
HTT: hyalinising trabecular thyroid tumor
LT: lymphocytic thyroiditis
MTC: medullary thyroid cancer
PDTC: poorly differentiated thyroid cancer
PSMA: prostate specific membrane antigen
PTC: papillary thyroid cancer
SNG: sporadic nodular goiter
UT: unspecific thyroiditis
