# Supplementary material for: Neovascular PSMA expression is a common feature in malignant neoplasms of the thyroid
Source: Oncotarget. 2018 Jan 4;9(11):9867–74. doi: 10.18632/oncotarget.23984 (PMC5839407; doi:10.18632/oncotarget.23984)
Supplement: Supplementary file 1 [file oncotarget-09-9867-s001.pdf]

Neovascular PSMA expression is a common feature in malignant neoplasms of the thyroid

SUPPLEMENTARY MATERIALS

Supplementary Table 1: Semi-quantitative immunohistochemistry results. See_Supplementary_ Table 1
