# Supplementary material for: Neovascular PSMA expression is a common feature in malignant neoplasms of the thyroid
Source: Oncotarget. 2018 Jan 4;9(11):9867–74. doi: 10.18632/oncotarget.23984 (PMC5839407; doi:10.18632/oncotarget.23984)
Supplement: Supplementary file 2 [file oncotarget-09-9867-s002.docx]

**Supplementary Table 1:** Semi-quantitative immunohistochemistry results

| **No.** | **Case** | **PSMA Immunohistochemistry** | | | | | | | | |
| --- | --- | --- | --- | --- | --- | --- | --- | --- | --- | --- |
|  |  | **(Neo)vasculature proportion [%]** | **(Neo)vasculature intensity* [0-3]** | **Labelling index**** |  | **(Tumor) cells proportion [%]** | **(Tumor) cells intensity*[0-3]** | **Labelling index**** |  |  |
| 1 | PTC | 80 | 3 | 2 |  | 0 | 0 | 0 |  |  |
| 2 | PTC | 50 | 3 | 2 |  | 0 | 0 | 0 |  |  |
| 3 | PTC | 10 | 3 | 2 |  | 0 | 0 | 0 |  |  |
| 4 | PTC | 10 | 3 | 2 |  | 0 | 0 | 0 |  |  |
| 5 | PTC | 10 | 3 | 2 |  | 0 | 0 | 0 |  |  |
| 6 | PTC | 25 | 3 | 2 |  | 0 | 0 | 0 |  |  |
| 7 | PTC | 20 | 3 | 2 |  | 0 | 0 | 0 |  |  |
| 8 | PTC | 10 | 3 | 2 |  | 0 | 0 | 0 |  |  |
| 9 | PTC | 10 | 3 | 2 |  | 0 | 0 | 0 |  |  |
| 10 | PTC | <5 | 1 | 1 |  | 0 | 0 | 0 |  |  |
| 11 | PTC | <5 | 1 | 1 |  | 0 | 0 | 0 |  |  |
| 12 | PTC | <5 | 1 | 1 |  | 0 | 0 | 0 |  |  |
| 13 | PTC | <5 | 2 | 1 |  | 0 | 0 | 0 |  |  |
| 14 | PTC | <5 | 2 | 1 |  | 0 | 0 | 0 |  |  |
| 15 | PTC | <5 | 2 | 1 |  | 0 | 0 | 0 |  |  |
| 16 | PTC | <5 | 2 | 1 |  | 0 | 0 | 0 |  |  |
| 17 | PTC | <5 | 2 | 1 |  | 0 | 0 | 0 |  |  |
| 18 | PTC | <5 | 1 | 1 |  | 0 | 0 | 0 |  |  |
| 19 | PTC | 0 | 0 | 0 |  | 0 | 0 | 0 |  |  |
| 20 | PTC | 0 | 0 | 0 |  | 0 | 0 | 0 |  |  |
| 21 | PTC | 0 | 0 | 0 |  | 0 | 0 | 0 |  |  |
| 22 | PTC | 0 | 0 | 0 |  | 0 | 0 | 0 |  |  |
| 23 | PTC | 0 | 0 | 0 |  | 0 | 0 | 0 |  |  |
| 24 | PTC | 0 | 0 | 0 |  | 0 | 0 | 0 |  |  |
| 25 | PTC | 0 | 0 | 0 |  | 0 | 0 | 0 |  |  |
| 26 | PTC | 0 | 0 | 0 |  | 0 | 0 | 0 |  |  |
| 27 | PTC | 0 | 0 | 0 |  | 0 | 0 | 0 |  |  |
| 28 | PTC | 0 | 0 | 0 |  | 0 | 0 | 0 |  |  |
| 29 | PTC | 0 | 0 | 0 |  | 0 | 0 | 0 |  |  |
| 30 | PTC | 0 | 0 | 0 |  | 0 | 0 | 0 |  |  |
| 31 | PTC | 0 | 0 | 0 |  | 0 | 0 | 0 |  |  |
| 32 | FTC | 50 | 3 | 2 |  | 0 | 0 | 0 |  |  |
| 33 | FTC | 5 | 3 | 2 |  | 0 | 0 | 0 |  |  |
| 34 | FTC | <5 | 2 | 1 |  | 0 | 0 | 0 |  |  |
| 35 | FTC | <5 | 2 | 1 |  | 0 | 0 | 0 |  |  |
| 36 | FTC | 0 | 0 | 0 |  | 0 | 0 | 0 |  |  |
| 37 | FTC | 0 | 0 | 0 |  | 0 | 0 | 0 |  |  |
| 38 | FTC | 0 | 0 | 0 |  | 0 | 0 | 0 |  |  |
|  |  |  |  |  |  |  |  |  |  |  |

| **No.** | **Case** | **PSMA Immunohistochemistry** | | | | | | | | |
| --- | --- | --- | --- | --- | --- | --- | --- | --- | --- | --- |
|  |  | **(Neo)vasculature proportion [%]** | **(Neo)vasculature intensity* [0-3]** | **Labelling index**** |  | **(Tumor) cells proportion [%]** | **(Tumor) cells intensity*[0-3]** | **Labelling index**** |  |  |
| 39 | FTC | 0 | 0 | 0 |  | 0 | 0 | 0 |  |  |
| 40 | FTC | 0 | 0 | 0 |  | 0 | 0 | 0 |  |  |
| 41 | FTC | 0 | 0 | 0 |  | 0 | 0 | 0 |  |  |
| 42 | MTC | <5 | 2 | 1 |  | 0 | 0 | 0 |  |  |
| 43 | MTC | <5 | 2 | 1 |  | 0 | 0 | 0 |  |  |
| 44 | MTC | <5 | 1 | 1 |  | 0 | 0 | 0 |  |  |
| 45 | MTC | <5 | 1 | 1 |  | 0 | 0 | 0 |  |  |
| 46 | MTC | 0 | 0 | 0 |  | 0 | 0 | 0 |  |  |
| 47 | MTC | 0 | 0 | 0 |  | 0 | 0 | 0 |  |  |
| 48 | MTC | 0 | 0 | 0 |  | 0 | 0 | 0 |  |  |
| 49 | MTC | 0 | 0 | 0 |  | 0 | 0 | 0 |  |  |
| 50 | MTC | 0 | 0 | 0 |  | 0 | 0 | 0 |  |  |
| 51 | MTC | 0 | 0 | 0 |  | 0 | 0 | 0 |  |  |
| 52 | MTC | 0 | 0 | 0 |  | 0 | 0 | 0 |  |  |
| 53 | MTC | 0 | 0 | 0 |  | 0 | 0 | 0 |  |  |
| 54 | PDTC | 10 | 3 | 2 |  | 0 | 0 | 0 |  |  |
| 55 | PDTC | 5 | 3 | 2 |  | 0 | 0 | 0 |  |  |
| 56 | PDTC | 10 | 3 | 2 |  | 0 | 0 | 0 |  |  |
| 57 | PDTC | 60 | 3 | 2 |  | 0 | 0 | 0 |  |  |
| 58 | PDTC | <5 | 2 | 1 |  | 0 | 0 | 0 |  |  |
| 59 | PDTC | <5 | 1 | 1 |  | 0 | 0 | 0 |  |  |
| 60 | DTC | 10 | 3 | 2 |  | 0 | 0 | 0 |  |  |
| 61 | DTC | 10 | 3 | 2 |  | 0 | 0 | 0 |  |  |
| 62 | DTC | 60 | 3 | 2 |  | 0 | 0 | 0 |  |  |
| 63 | DTC | 30 | 2 | 2 |  | 0 | 0 | 0 |  |  |
| 64 | SNG | 0 | 0 | 0 |  | 0 | 0 | 0 |  |  |
| 65 | SNG | 0 | 0 | 0 |  | 0 | 0 | 0 |  |  |
| 66 | SNG | 0 | 0 | 0 |  | 0 | 0 | 0 |  |  |
| 67 | SNG | 0 | 0 | 0 |  | 0 | 0 | 0 |  |  |
| 68 | SNG | 0 | 0 | 0 |  | 0 | 0 | 0 |  |  |
| 69 | SNG | 0 | 0 | 0 |  | 0 | 0 | 0 |  |  |
| 70 | SNG | 0 | 0 | 0 |  | 0 | 0 | 0 |  |  |
| 71 | SNG | 0 | 0 | 0 |  | 0 | 0 | 0 |  |  |
| 72 | SNG | 0 | 0 | 0 |  | 0 | 0 | 0 |  |  |
| 73 | SNG | 0 | 0 | 0 |  | 0 | 0 | 0 |  |  |
| 74 | SNG | 0 | 0 | 0 |  | 0 | 0 | 0 |  |  |
| 75 | SNG | 0 | 0 | 0 |  | 0 | 0 | 0 |  |  |
| 76 | SNG | 0 | 0 | 0 |  | 0 | 0 | 0 |  |  |
| 77 | SNG | 0 | 0 | 0 |  | 0 | 0 | 0 |  |  |
| 78 | SNG | 0 | 0 | 0 |  | 0 | 0 | 0 |  |  |
| 79 | SNG | 0 | 0 | 0 |  | 0 | 0 | 0 |  |  |
|  |  |  |  |  |  |  |  |  |  |  |
| **No.** |  | **PSMA Immunohistochemistry** | | | | | | | | |
|  |  | **(Neo)vasculature proportion [%]** | **(Neo)vasculature intensity* [0-3]** | **Labelling index**** |  | **(Tumor) cells proportion [%]** | **(Tumor) cells intensity*[0-3]** | **Labelling index**** |  |  |
| 80 | SNG | 0 | 0 | 0 |  | 0 | 0 | 0 |  |  |
| 81 | SNG | 0 | 0 | 0 |  | 0 | 0 | 0 |  |  |
| 81 | FA | 50 | 3 | 2 |  | 0 | 0 | 0 |  |  |
| 83 | FA | <5 | 2 | 1 |  | 0 | 0 | 0 |  |  |
| 84 | FA | 0 | 0 | 0 |  | 0 | 0 | 0 |  |  |
| 85 | FA | 0 | 0 | 0 |  | 0 | 0 | 0 |  |  |
| 86 | FA | 0 | 0 | 0 |  | 0 | 0 | 0 |  |  |
| 87 | FA | 0 | 0 | 0 |  | 0 | 0 | 0 |  |  |
| 88 | FA | 0 | 0 | 0 |  | 0 | 0 | 0 |  |  |
| 89 | FA | 0 | 0 | 0 |  | 0 | 0 | 0 |  |  |
| 90 | FA | 0 | 0 | 0 |  | 0 | 0 | 0 |  |  |
| 91 | HTT | <5 | 2 | 1 |  | 0 | 0 | 0 |  |  |
| 92 | HTT | 0 | 0 | 0 |  | 0 | 0 | 0 |  |  |
| 93 | GD | <5 | 1 | 0 |  | 0 | 0 | 0 |  |  |
| 94 | GD | 0 | 0 | 0 |  | 0 | 0 | 0 |  |  |
| 95 | GD | 0 | 0 | 0 |  | 0 | 0 | 0 |  |  |
| 96 | LT | <5 | 2 | 1 |  | 0 | 0 | 0 |  |  |
| 97 | LT | 0 | 0 | 0 |  | 0 | 0 | 0 |  |  |
| 98 | GT | 0 | 0 | 0 |  | 0 | 0 | 0 |  |  |
| 99 | UT | 0 | 0 | 0 |  | 0 | 0 | 0 |  |  |
| 100 | UT | 0 | 0 | 0 |  | 0 | 0 | 0 |  |  |
| 101 | UT | 0 | 0 | 0 |  | 0 | 0 | 0 |  |  |
|  |  |  |  |  |  |  |  |  |  |  |

*** intensity staining:**negative (0), weak (1 = barely perceptible staining at high power (400x) magnification), moderate (2 = readily apparent at low power (40x) magnification) or strong (3)

**** labelling index:**A weak (1) or moderate (2) staining intensity in < 5% of the neovasculature and a weak (1) staining intensity in > 5% of the neovasculature was allocated to the “low expression” group (PSMA labelling index = 1), whereas a moderate (2) staining intensity in > 5% of the neovasculature and a strong (3) staining intensity in < or > 5% of the neovasculature were assigned to the “strong expression” group (PSMA labelling index = 2).

**Abbreviations:**PTC, papillary thyroid cancer; FTC: follicular thyroid cancer; MTC, medullary thyroid cancer; PDTC, poorly differentiated thyroid cancer; DTC, dedifferentiated (anaplastic) thyroid cancer; SNG, sporadic nodular goiter; FA, follicular adenoma; HTT, hyalinising trabecular thyroid tumor; GD, Grave´s Disease; LT, lymphocytic thyroiditis; GT, granulomatous thyroiditis; UT, unspecific thyroiditis.
